# The role of perioperative sodium bicarbonate infusion affecting renal function after cardiothoracic surgery

**DOI:** 10.3389/fphar.2014.00127

**Published:** 2014-06-02

**Authors:** Katja R. Turner, Edward C. Fisher, Erinn M. Hade, Timothy T. Houle, Michael V. Rocco

**Affiliations:** ^1^Department of Anesthesiology, Wexner Medical Center at the Ohio State UniversityColumbus, OH, USA; ^2^Metrolina Nephrology AssociatesCharlotte, NC, USA; ^3^Center for Biostatistics, The Ohio State UniversityColumbus, OH, USA; ^4^Department of Anesthesia, Wake Forest School of MedicineWinston-Salem, NC, USA; ^5^Section on Nephrology, Department of Medicine, Wake Forest School of MedicineWinston-Salem, NC, USA

**Keywords:** acute kidney injury, cardiovascular surgery, bicarbonate therapy

## Abstract

Cardiac surgery associated acute kidney injury (CSA-AKI) is associated with poor outcomes including increased mortality, length of hospital stay (LOS) and cost. The incidence of acute kidney injury (AKI) is reported to be between 3 and 30% depending on the definition of AKI. We designed a multicenter randomized controlled trial to test our hypothesis that a perioperative infusion of sodium bicarbonate (SB) during cardiac surgery will attenuate the post-operative rise in creatinine indicating renal injury when compared to a perioperative infusion with normal saline. An interim analysis was performed after data was available on the first 120 participants. A similar number of patients in the two treatment groups developed AKI, defined as an increase in serum creatinine the first 48 h after surgery of 0.3 mg/dl or more. Specifically 14 patients (24%) who received sodium chloride (SC) and 17 patients (27%) who received SB were observed to develop AKI post-surgery, resulting in a relative risk of AKI of 1.1 (95% CI: 0.6–2.1, chi-square *p*-value = 0.68) for patients receiving SB compared to those who received SC. The data safety monitoring board for the trial recommended closing the study early as there was only a 12% probability that the null hypothesis would be rejected. We therefore concluded that a perioperative infusion of SB failed to attenuate the risk of CSA-AKI.

## Introduction

Cardiac surgery associated acute kidney injury (CSA-AKI) is described as an acute worsening in kidney function observed after cardiac surgery that is associated with poor outcomes (Bellomo et al., 2008). Increased mortality, length of hospital stay (LOS) and cost are all more likely in this setting (Chertow et al., 1998; Lassnigg et al., 2004; Loef et al., 2005). The incidence of acute kidney injury (AKI) is reported to be between 3 and 30% depending on the definition of AKI. AKI requiring renal replacement therapy, occurring in 1–5%, has a reported mortality rate ranging from 60 to 90% (Frost et al., 1991; Chertow et al., 1998; Mangano et al., 1998; Loef et al., 2005; Robert et al., 2010). Recent publications reported increased 30 day mortality for even mild levels of acute renal failure (ARF) which do not require dialysis (Lassnigg et al., 2004; Loef et al., 2005). Despite advances in medications, dialysis techniques and treatment strategies, the incidence, morbidity and mortality associated CSA-AKI has not improved significantly in 40 years. The pathophysiology of AKI is multifold. Processes likely to contribute are toxins, metabolic factors, ischemia-reperfusion injury, neurohormonal activation, inflammation, and generation of reactive oxygen species (McCord, 1985; Andersson et al., 1994; Lema et al., 1995; Rinder et al., 2003; Doi et al., 2004; McCullough et al., 2006; Arora et al., 2008; Ascione et al., 2008; Filsoufi et al., 2008; Greilich et al., 2008; Lecomte et al., 2008; Fontaine et al., 2009). The prevention of AKI in this setting is clinically and economically an important goal for CV surgery and anesthesia programs. The multitude of causes makes it more difficult to find a preventive intervention.

Preventive techniques for perioperative AKI have traditionally focused on maintenance of hemodynamic stability, repletion of blood and electrolyte imbalances and avoidance of nephrotoxic drugs. Patients presenting for cardiovascular surgery were found to be at increased risk for AKI. Several scoring systems have been developed to stratify patients at increased risk (Higgins et al., 1992; Andersson et al., 1993; Chertow et al., 1997; Fortescue et al., 2000; Brown et al., 2007). Few studies have examined preventive strategies for AKI in this setting. In a 1995 meta-analysis, Conger reviewed all previous studies evaluating pharmacologic, dialytic, and nutritional attempts at preventing ischemic ARF in a variety of clinical settings (Conger, 1995). Commonly used therapies such as furosemide, dopamine, and mannitol were found to offer no significant reduction in rates of ARF (Conger, 1995; Bellomo et al., 2000). Despite the lack of beneficial effects, an Austrian study in 2000 revealed that 25–35% of all cardiovascular surgery cases in 38 European hospitals were using furosemide and low dose Dopamine empirically (Lassnigg et al., 2000). Recent preventive attempts included Fenoldopam, natriuretic peptides, hypertonic saline, and immunomodulation revealing limited benefit with few human studies included in those analyses (Conger, 1995; Allgren et al., 1997; Block and Manning, 2002; Garwood et al., 2003).

The renal medulla receives a disproportionate share of renal blood flow (<10% medulla vs. ~90% cortex), making it an environment of relative hypoxia and acidosis, where the urine first becomes acidic. This region includes many of the active transport pumps which require ATP to achieve maintenance of the renal osmotic gradient. For these reasons it is the medulla which is often the initial site of renal injury in perioperative settings involving ischemic acute tubular necrosis (ATN) (Myers and Moran, 1986; Brezis and Epstein, 1993). Cardiovascular surgery, especially when utilizing cardiopulmonary bypass, is considered to be a high risk procedure for the development of AKI. Possible etiologies include hypoperfusion after initiating cardiopulmonary bypass, ischemia-reperfusion injury, activation of inflammatory pathways, and reduction in oxygen supply due to hemodilution. Hemodilution and the decline in perfusion pressure may further aggravate oxygen delivery and medullary hypoxia. Protecting the medulla by improving blood flow, oxygenation, and limiting oxidative stress by free radical formation are important goals. Sodium bicarbonate (SB), a slightly alkaline solution, has intrinsic natriuretic and diuretic effects in addition to its ability to alkalinize tubular fluid (Atkins, 1986). It is our thought that these properties contribute to increase oxygen delivery while reducing free radical formation due to neutralizing acidosis in this vulnerable region of the kidney. These properties make SB a novel anion in place of more traditional fluids used in the perioperative period. In a pilot study, we had found that SB *appears to* protect the kidney from ischemic injury in the setting of cardiovascular surgery. Rates of AKI, particularly in high risk patients, were reduced using prophylactic bicarbonate based infusions in place of traditional maintenance fluids (Fisher et al., 2004, 2005).

We hypothesized that a perioperative infusion of SB will attenuate the post-operative rise in creatinine indicating renal injury. The primary outcome, AKI, was determined based on post-operative changes in creatinine values measured in the bicarbonate group vs. normal saline group. The maximum change in glomerula filtration rate (GFR), LOS, incidence of dialysis, and mortality rate were assessed as secondary outcomes.

## Methods

### Design

This was a multicenter, randomized, controlled trial of IV SB vs. IV normal saline in high risk adult patients requiring cardiovascular surgery to test the hypothesis that SB would be protective against AKI post-operatively. We developed our inclusion criteria based on studies describing patients at risk for developing severe renal dysfunction after requiring cardiovascular surgery (Higgins et al., 1992; Andersson et al., 1993; Chertow et al., 1997, 1998; Fortescue et al., 2000; Thakar et al., 2005; Brown et al., 2007). Inclusion criteria included planned receipt of cardiovascular surgery as well as increased risk factors for AKI post-operatively, including either an estimated GFR of <60 ml/min/1.73 meters squared or the combination of age greater than 70 years and complex surgery (as defined in Table 1). Exclusion criteria included eGFR of <15 ml/min, emergency surgery, distal aortic surgery, and pre-operative bicarbonate level >30 mEq/l.

**Table 1:** **Inclusion and exclusion criteria**.

**Inclusion criteria**	
• Calculated GFR ≤ 60 ml/min/m^2^ (MDRD) (OR)	
• Any combination of two (2) of the following	
• Age ≥ 70	
• Complex surgery (any of the following)	
- CABG/Valve	- History of PVD surgery
- Redo operation	- EF < 35%
- Deep hypothermic arrest	- DM
- Prior kidney transplant	- ≥ 2 valves
- Proximal Aortic surgery (i.e., ascending aortic aneurysm)	
**Exclusion criteria**	
• Age < 18	
• Pre-existing ESRD (dialysis patients)	
• Pre-op GFR ≤ 15 ml/min/m^2^	
• Pre-op bicarbonate level ≥ 30 mEq/L	
• Emergency surgery (unable to effectively consent)	
• Pregnancy	
• Heart transplant (OHT)	
• Distal Aortic surgery (i.e., descending aortic aneurysm)	
• Procedure does not require central venous access	

*CABG, Coronary Artery Bypass Graft; DM, Diabetes Mellitus; EF, Ejection Fraction; ESRD, End Stage Renal Disease; GFR, Glomerular Filtration Rate; MDRD, Modification of Diet in Renal Disease Study; OHT, Orthotopic Heart Transplantation*.

Patients undergoing cardiovascular surgery who met the inclusion and exclusion criteria were identified pre-operatively by study personnel who explained the study protocol and obtained written informed consent for the trial. Patients eligible for the study were identified more than 12 h prior to elective or urgent surgery to allow enough time to make an informed decision regarding the trial. Qualifying patients were randomized to receive either SB or normal saline (controls) as perioperative infusions. Subjects enrolled in the trial received a weight based pre-operative bolus of the designated fluid followed post-operatively by a weight based infusion of the designated fluid for 22 h. Serum creatinine levels were obtained pre-operatively and then on a daily basis for at least 72 h post-operatively. The serum creatinine level was measured daily using the Jaffe method. Other data collected during the trial period included length of stay in the hospital and need for dialytic therapy for treatment of AKI during hospitalization. The protocol for this prospective randomized controlled multicenter trial was approved by the Wake Forest and Ohio State School of Medicine's Institutional Review Boards and registered with Clinical trials.gov (NCT0048435).

### Surgical technique

All surgical procedures were performed through a median sternotomy. Equipment used during cardiopulmonary bypass included: a roller pump system (Terumo system I) with trillium coated tubing (Medtronic), a membrane oxygenator (Affinity trillium coated, Medtronic's), and an arterial filter (351 20 micron by Medtronic). The circuit was primed with Plasmalyte A, as well as 10,000 units of unfractionated heparin, 25 mg of Mannitol, and 50 mEq SB with a final pump volume of ~700 ml. The patients were resuscitated with crystalloids (lactated ringer's or normal saline solutions), blood products or colloids (albumin 5%). The patient's maximum blood glucose levels were maintained at <190 mg/dl using insulin infusions ± boluses as deemed necessary. Throughout the procedure, the patients' anesthetic was maintained using volatile anesthetics, benzodiazepines, and narcotics (fentanyl, morphine, and methadone) as per anesthesiologists' preference. Perioperative hemodynamic management targeted either the baseline cardiac output (CO) prior to induction or a CO >2.1 l/min/m2 and a mean arterial blood pressure >60 mmHg. The intraoperative fluid management was directed by the treating anesthesiologist and documented. All clinical data was collected prospectively and entered into a computerized database by the research team.

### Administration of IV bicarbonate and IV normal saline

Those patients allocated to the normal saline arm (controls) underwent a protocol consisting of an initial bolus consisting of 0.154 M NaCl (NS) at 5.0 ml/kg (or a maximum bolus dose of 500 ml to avoid excessive fluid overload) given over 15 min prior to initiation of cardio-pulmonary bypass. Following the bolus, these patients were infused with NS (0.154 M) at a rate of 1.0 ml/kg/h (or a maximum rate of infusion of 125 mL/min to avoid excessive fluid overload) for a total of 10 h, and decreased to 0.4 ml/kg/h for a remaining 12 h; essentially until the following day. Those patients allocated to the SB (NaHCO_3_) arm received a 0.150 M NaHCO_3_ solution prepared in a sterile water base. Bicarbonate solution was prepared by a central pharmacy at each site. Blinding of clinical staff to treatment arm was not performed as the data collected in the study was objective data. This protocol consisted of an initial bolus of 0.150 M NaHCO_3_ at 5.0 ml/kg (or a maximum bolus dose of 500 ml) given prior to initiation of cardio-pulmonary bypass, followed by a 0.150 M NaHCO_3_ infusion at a rate of 1.0 ml/kg/h (or a maximum rate of infusion of 125 mL/min) for a total of 10 h followed by a NaHCO_3_ infusion of 0.4 ml/kg/h for another 12 h, as was done with the NS treated patients. The goal bicarbonate dose in this study was ~3.0 mEq/kg. This dose was based on what our group found to be efficacious in our previous studies (Fisher et al., 2004, 2005). The study protocol included mechanisms to adjust the bicarbonate infusion rates based on post-operative lab values. The bicarbonate infusion rate was decreased by one half (1/2) for all patients whose serum bicarbonate level was greater than or equal to 30 mmol/L post-operatively. This half dose infusion was continued until the serum bicarbonate returned to a level less than 30 mmol/L at which time the rate was increased back to the previous level. For any patient with a post-operative serum bicarbonate level greater than or equal to 35 mmol/ml the infusion rate was decreased to 10 mL per hour (KVO) until the bicarbonate level dropped to less than 30 mmol/ml at which time the infusion rate was increased back to previous levels.

This protocol consisted of ~22 h of IV study infusion. Any other IV fluid that the respective surgeons or anesthesiologists deemed necessary was allowed as part of routine patient management. For those patients felt to be volume overloaded, the randomized fluid was reduced to 10 mL per hour until the clinical conditions allow the higher rate to be resumed.

### Analytic plan

The primary outcome of the trial was to determine the rate of AKI in the study population. The study sample size was determined based on previous information in a high risk population. Historic data reported rates of ARF, defined arbitrarily as a change in SCr ≥ 1.0 mg/dL, to be ~11.7% in patients treated with sodium chloride (SC) and 2.7% in patients treated with the bicarbonate solution (Fisher et al., 2004). To observe these differences between treatment groups in ARF with 90% power and a 5% type one error rate, ~360 patients (180 in each randomized group) would need to be enrolled in the trial.

After the study was initiated, a consensus definition of AKI was introduced in 2004 (RIFLE) and subsequently modified based on the AKIN staging system (Mehta et al., 2007). This system defines stage 1 of AKI as a 1.5 fold or ≥0.3 mg/dl increase in the serum creatinine level from baseline. After the initiation of the trial the AKIN staging system had become a clearly defined reference for AKI and in consultation with our DSMB, but prior to data analysis, the AKIN definition of stage I AKI was chosen as the primary means to determine the presence of AKI. For analytic purposes, we evaluated our results based on three definitions of ARF: ARF defined as an increase in serum creatinine (SCr) by 1 mg/dl in 72 h, ARF (AKI) defined as an increase of 25% in SCr, and ARF (AKI) defined as an increase in SCr of 0.3 mg/dl.

Secondary endpoints of interest include the maximum change in estimated serum creatinine and the glomerular filtration rate (GFR) during the first 72 h post-operatively, LOS, incidence of dialysis (HD) and mortality.

### Interim analysis and determination of futility

At the interim time, we tested the hypothesis of a difference in the proportion of patients with any AKI between treatment groups, as described by the AKIN staging system (AKI as a 1.5 fold or ≥0.3 mg/dl increase in serum creatinine from baseline). Data from 120 patients was available for analysis (2 patients did not have post-surgery creatinine measurements recorded at OSU—one in each treatment arm). AKI was observed in 31 (26%) of patients, with nearly equal numbers in each randomized group [14 in the normal saline (24%), 17 in the SB group (27%)], resulting in an interim test *p*-value (chi-square test) of 0.95. Given this information, we could clearly state that early efficacy was not met. In fact based on this information, conditional power based on the effect size determined with the 120 patients accrued, was estimated to be only 12%. Given this very low estimated conditional power, we, along with our data and safety monitoring committee, recommended closing the trial and reporting on the current study.

### Primary and secondary outcome analysis

The primary analysis of AKI rates between treatment groups was evaluated by the chi-squared test and included estimates of the relative risk and associated 95% confidence intervals. Secondary outcomes included length of stay and maximum change in GFR which were compared by a two-sample *t*-test (without assuming equal variances) or by the chi-square test for categorical outcomes such as mortality and incidence of dialysis. All tests were two-sided and considered significant at the 0.05 level. Analyses and data management were performed using SAS software, Version 9.2 of the SAS System. Copywrite, SAS Institute Inc., Cary, NC, USA.

## Results

### Acute kidney injury and other renal outcomes

Patients were recruited to the study between August 2006 and February 2011. Patient characteristics of the study population are shown in Table 2. The average patient age was ~70 years and the majority of the participants were Caucasians. The type of surgery performed included coronary artery bypass grafting (alone or in combination with valvular surgery), valvular surgery, aortic (ascending and aortic arch) as well as placement of ventricular assist devices.

**Table 2:** **Patient demographics and pre-surgery clinical characteristics**.

**Demographics**	**Sodium chloride (*n* = 59)**	**Sodium bicarbonate (*n* = 64)**
Age, years^*^	69.7 (13.5)	70.2 (12.6)
Sex		
Female	16 (27%)	30 (47%)
Male	43 (73%)	34 (53%)
Race		
White	47 (80%)	57 (89%)
Black	9 (15%)	5 (8%)
Unknown	3 (5%)	2 (3%)
Weight, kg^*^^a^	90.2 (19.5)	85.4 (18.4)
Site		
OSU	36 (61%)	35 (55%)
Wake Forest	23 (39%)	29 (45%)
**MEDICAL HISTORY AND COMORBIDITIES**		
Previous CV surgery	13 (22%)	22 (34%)
Previous PVD surgery	5 (8%)	7 (11%)
History of kidney disease	2 (3%)	0 (0%)
History of HTN	51 (86%)	51 (80%)
History of DM	26 (44%)	30 (47%)
History of COPD	6 (10%)	12 (19%)
History of MI	13 (22%)	10 (16%)
**MEDICATION**		
Aprotinin	14 (24%)	16 (25%)
Amicar	18 (31%)	13 (20%)
Contrast	30 (51%)	34 (53%)
**MEASURES OF KIDNEY FUNCTION**		
Plasma creatinine mg/dl^*^^b^	1.2 (0.5)	1.0 (0.4)
Plasma HCO_3_^*^^c^	25.8 (3.1)	26.1 (3.0)
Plasma CA^*^^d^	8.2 (1.8)	8.2 (1.8)
Plasma K^*^^e^	4.3 (0.4)	4.2 (0.4)

*COPD, Chronic Obstructive Pulmonary Disease; DM, Diabetes Mellitus; HTN, Hypertension; MI, Miocardial Infarction; PVD, Peripheral Vascular Disease*.

* Mean (standard deviation);

a available on 59 (SC) and 63 (SB);

b available on 57 (SC) and 62 (SB);

c available on 55 (SC) and 61 (SB);

d available on 48 (SC) and 57 (SB);

e *available on 57 (SC) and 61 (SB)*.

Due to slow enrollment, the study team and independent clinical ethicist agreed to perform an interim analysis once 120 subjects were enrolled to determine if treatment efficacy had been met. A total of 123 patients were enrolled between the two centers to either receive intravenous SB (*n* = 64) or SC (*n* = 59) infusions. As described earlier, accrual to this study was stopped early due to no evidence of efficacy (See Interim analysis). Data from 120 patients were available for analysis (2 patients did not have post-surgery creatinine measurements recorded at OSU;–one in each treatment arm). One patient suffered from infiltration of SB around the peripheral intravenous access site. The infiltration was treated, and the patient dropped out of the study.

A similar number of patients in the two treatment groups developed AKI, defined as an increase in serum creatinine the first 48 h after surgery of 0.3 mg/dl or more. Specifically 14 patients (24%) who received SC and 17 patients (27%) who received SB were observed to develop AKI post-surgery, resulting in a relative risk of AKI of 1.1 (95% CI: 0.6–2.1, chi-square *p*-value = 0.68) for patients receiving SB compared to those who received SC. AKI in the first 72 h occurred in 42 patients [22 patients (38%) in the SC group and 20 patients (32%) in the SB group] resulting in an estimated relative risk of 0.9 (95% CI: 0.5–1.4). AKI defined as an increase of 25% or more in serum creatinine (AKI25) in the first 24 or 48 h was also not substantially different between groups (Table 3). Moreover, ARF defined in the original study design, was not improved post-surgery by the use of SB. ARF was not frequently observed; only 3 patients (5%) in the SC group and 7 patients (11%) in the SB group had ARF (*RR* = 2.2; 95% CI: 0.6–8.0). A sub analysis of patients who had contrast during surgery, did not provided a substantial change to these conclusions concerning differences in AKI or ARF post-surgery between these groups.

**Table 3:** Outcomes by treatment group^*^.

	**Sodium chloride (*n* = 59)**	**Sodium bicarbonate (*n* = 64)**	**Relative risk (95% CI)**
**PRIMARY ENDPOINT**			
**AKI 0.3 mg/dl (72 h)**			
No	36 (62%)	42 (68%)	1.0
Yes	22 (38%)	20 (32%)	0.9 (0.5–1.4)
**SECONDARY ENDPOINTS**			
**ARF 1.0 mg/dl (72 h)**			
No	55 (95%)	55 (89%)	1.0
Yes	3 (5%)	7 (11%)	2.2 (0.6–8.0)
**AKI 0.3 mg/dl (48 h)**			
No	44 (76%)	45 (73%)	1.0
Yes	14 (24%)	17 (27%)	1.1 (0.6–2.1)
**AKI 25% change (48 h)**			
No	47 (81%)	44 (71%)	1.0
Yes	11 (19%)	18 (29%)	1.5 (0.8–3.0)
**AKI 25% change (72 h)**			
No	38 (66%)	41 (66%)	1.0
Yes	20 (34%)	21 (34%)	0.9 (0.6–1.6)
**Hemodialysis**			
No	50 (91%)	58 (94%)	1.0
Yes	5 (9%)	4 (6%)	0.7 (0.2–2.5)
**Death**			
No	48 (83%)	54 (86%)	1.0
Yes	10 (17%)	9 (14%)	0.8 (0.4–1.9)
**Length of Stay**			
Mean (std)	14.6 (14.4)	14.2 (15.4)	0.43 (–5.1–5.9)
**SUBSET WITH CONTRAST**			
**AKI 0.3 mg/dl (48 h)**			
No	21 (75.0)	22 (66.7)	1.0
Yes	7 (25.0)	11 (33.3)	1.3 (0.6–3.0)
**AKI 0.3 mg/dl (72 h)**			
No	19 (67.9)	22 (66.7)	1.0
Yes	9 (32.1)	11 (33.3)	1.0 (0.5–2.1)
**AKI 25% change (48 h)**			
No	23 (82.1)	21 (63.6)	1.0
Yes	5 (17.9)	12 (36.4)	2.0 (0.8–5.1)
**AKI 25% change (72 h)**			
No	20 (71.4)	21 (63.6)	1.0
Yes	8 (28.6)	12 (36.4)	1.3 (0.6–2.7)
**ARF 1.0 mg/dl (72 h)**			
No	27 (96.4)	29 (87.9)	1.0
Yes	1 (3.6)	4 (12.1)	3.4 (0.4–28.7)

*AKI, Acute Kidney Injury; ARF, Acute Renal Failure*.

* *Outcomes missing on 3 individuals (1 in the SC group and 2 in the SB group), except for death where it is missing in 2 individuals (1 in each group)*.

### Other outcomes

We observed a similar number of deaths (10 in the SC group and 9 in the SB group) and patients in need for post-operative dialysis (5 in the SC group and 4 in the SB group) in both groups. Further, the number of days spend in the hospital did not differ substantially between the treatment groups with an average of 14.2 days [standard deviation (sd) = 15.4] in SB patients and 14.6 days (*sd* = 14.2) in SC patients (*t*-test assuming unequal variances *p*-value = 0.88).

## Discussion

In this trial, we did not find a benefit of IV SB administration in decreasing the rate of AKI compared to placebo. The incidence of AKI defined as an increase of creatinine by 0.3 mg/dl in our study was not different between study groups (24% in the SC group, and 24% in the SB group). Moreover, ARF, as defined in the original study design, was rare and not improved by the use of SB. The incidence of ARF observed in our study was lower than historical data indicated; only 3 patients (5%) in the SC group and 7 (11%) patents in the SB group had ARF compared to 11.7% reported. Based on our data and the definition of ARF stated above, we observed no beneficial effect of a perioperative SB infusion, but rather a suggestion of increased incidence of ARF. During the enrollment of our patients, a pilot study was published by Haase et al., indicating a beneficial effect of a perioperative infusion of SB to prevent an increase of serum creatinine after cardiac surgery (Haase et al., 2009). The authors defined acute renal dysfunction as an increase in plasma creatinine concentration of greater than 25% from baseline within the 1st 5 days after cardiopulmonary bypass. Considering a sample size of 100 patients, they observed the risk of developing acute renal dysfunction to be reduced by 20% when using a perioperative bicarbonate infusion. Due to slow enrollment and time for an interim analysis, we analyzed our data in 2011, showing no beneficial effect of a perioperative SB infusion. Having a comparable study population and protocol to Haase's study, we analyzed our data using the Haase's definition of acute renal dysfunction. In contrast to their study, we observed the change in creatinine over 72 h following cardio-pulmonary bypass, with no beneficial effect of the perioperative administration of SB (Table 3). In an attempt to explain the difference in outcome, we closely compared the study protocols of Haase's and our study. Both protocols targeted a high risk population, started the administration of the study solution as a bolus followed by a maintenance infusion prior to initiating cardio-pulmonary bypass, and continued the infusion into the post-operative period. The protocols differed in the bolus amount (0.5 vs. 0.75 mmol/kg), and time administered (60 vs. 20 min), and the total amount of SB (4 vs. 3 mmol/kg), as well as the total amount of fluid administered (1250 ml regardless of weight vs. up to 2000 ml based on weight). Our study observed changes for 72 h following surgery, whereas Haase et al. studied their patients for an extra 2 days. Whether any of these differences are responsible for the difference in outcome remains debatable.

Nevertheless, our results are more in line with the results of studies recently published, that were conducted in a larger patient population (Heringlake et al., 2012; Haase et al., 2013; McGuinness et al., 2013). Two of these studies are multicenter randomized trials including a total of 777 patients, while the third study is a prospective observational cohort study including 304 patients (Heringlake et al., 2012). Since both randomized controlled studies used the same study protocol as Haase's pilot study, it seems unlikely that our study protocol is solely responsible for our inability to show a beneficial effect. We considered the possibly that the standard of care changed over time, and that SB was more effective in a historical patient population, since the initial beneficial effects of SB were observed in contrast induced nephropathies (Motohiro et al., 2011; Ueda et al., 2011). We posited that the standard of care changed to delay cardiac surgery after exposure to contrast. We performed a sub analysis on our study population recently receiving contrast to see whether SB has any beneficial effect in this subset of patients. We identified 28 patients in the SC and 33 patients in the SB group. After applying the 3 definitions of AKI mentioned above, we did not find any benefit in SB administration, but rather a trend of increased likelihood to develop AKI (Table 3). The prospective observational study differs from other studies mentioned in targeting all patients presenting for cardiac surgery without preference for patients at risk to develop kidney injury post-operatively (Heringlake et al., 2012).

A clear limitation of our study is the number of patients that we eventually enrolled. Our initial sample size, based on an incidence of renal failure of 11.7%, was 360 patients. With any lack of benefit demonstrated in the interim analysis, we terminated our study early. While even larger studies could not demonstrate a beneficial effect of perioperative treatment with SB, Haase's most recent work observed a trend of increased mortality in patients receiving SB (Haase et al., 2013). They have therefore recommended abstaining from using SB infusions as prophylaxis to ameliorate CSA-AKI. Based on our and currently available findings, we cannot recommend the prophylactic use of a perioperative SB, or suggest the intervention to be tested on a larger study population. The most likely explanation for the lack of benefit of a perioperative SB infusion is the multifactorial etiology of CSA-AKI, and a modification of only one contributing factor insufficient to impact the development of renal failure.

In conclusion, patients undergoing cardio-vascular surgery exposed to cardiopulmonary bypass represent the second most common group of patients treated in intensive care unit settings to develop AKI (Uchino et al., 2005). Based on our previous experience, we tested the hypothesis that the administration of a prophylactic perioperative SB infusion will alter the development of AKI in a high risk population presenting for high risk surgery. We were unable to demonstrate a beneficial effect applying historic or recently revised definitions of AKI.

### Conflict of interest statement

This study was funded, in part, by a grant for the Southeastern Kidney Council.
